# Evaluation of the Regulatory Review Process in Zimbabwe: Challenges and Opportunities

**DOI:** 10.1007/s43441-020-00242-z

**Published:** 2021-01-02

**Authors:** Tariro Sithole, Gugu Mahlangu, Sam Salek, Stuart Walker

**Affiliations:** 1 School of Life and Medical Sciences, University of Hertfordshire, Hatfield, UK; 2 Medicines Control Authority of Zimbabwe, Harare, Zimbabwe; 3 Centre for Innovation in Regulatory Science, 160 Blackfriars Road, London, SE1 8EZ UK; 4 Institute of Medicines Development, Cardiff, UK

**Keywords:** Medicine control authority of Zimbabwe (MCAZ), International best practice, Regulatory review models, Good review practices, Timelines, Good decision-making practice

## Abstract

**Purpose:**

The aims of this study were to assess the current regulatory review process of the Medicines Control Authority of Zimbabwe (MCAZ), identify key milestones and target timelines, evaluate the overall performance from 2017 to 2019, identify good review practices, evaluate the quality of decision-making processes, and identify the challenges and opportunities for improvement.

**Methods:**

A questionnaire was completed by the MCAZ. The agency has participated in the Optimising Efficiencies in Regulatory Agencies (OpERA) program, a multinational endeavor to characterize assessment procedures and metrics associated with regulatory agencies and regional regulatory initiatives. Data identifying the milestones and overall approval times for all products registered MCAZ from 2017 to 2019 were collected and analyzed.

**Results:**

The MCAZ conducts a full review of quality, safety, and efficacy data for generics and biosimilars not approved by a reference agency, an abridged review for products approved by a reference agency and a verification review for World Health Organization prequalified products under the collaborative registration procedure. The highest number of reviewed products is generics manufactured by foreign companies. There has been an improvement in review times for all categories of products over the three-year period. Guidelines, standard operating procedures, and review templates are in place and the majority of indicators for good review practices are implemented. Although quality decision-making practices are implemented, there is no formal framework in place.

**Conclusion:**

The MCAZ successfully implements three types of review models in line with international standards. Overall, target timelines are realistic and what is achievable with the current available resources. Recommendations made such as the review of available human resources, separation of agency and company time when setting and measuring targets, review of the templates and benefit-risk framework used for abridged review, and development of a decision-making framework present opportunities for an enhanced regulatory review process.

## Introduction

### Zimbabwe and the National Medicines Regulatory Authority

Zimbabwe is a landlocked country with a gross domestic product (GDP) of 31 billion USD and a population of 14.5 million in 2018 [1]. The country is bordered by South Africa, Namibia, Zambia, Botswana, and Mozambique [2]. Regulation of medicines began in 1969 through an Act of Parliament, the Drugs and Allied Substances Control Act of 1969 (Chapter 15.03) [3]. The Medicines and Allied Substances Control Act was promulgated in 1997, creating an autonomous agency independent of the fiscus, the Medicines Control Authority of Zimbabwe (MCAZ). The MCAZ’s chemistry laboratory is prequalified by the World Health Organization [4]. The MCAZ has a robust quality management system, which resulted in the ISO 9001 certification by the Standards Association of Zimbabwe in 2019 [5]. The MCAZ offers training to regulators on the continent and as a result is designated as a Regional Centre of Regulatory Excellence (RCORE) for medicines evaluation and registration, clinical trials authorization, and quality assurance and control by the African Union’s Development Agency New Partnership for Africa Development (AUDA – NEPAD) [6]. In addition, the MCAZ is a founding member of the ZAZIBONA collaborative medicines registration initiative and also responsible for coordinating the Southern African Development Community (SADC) Medicines Registration Harmonization (MRH) project as the implementing agency [7]. The project aims to build the regulatory capacity of member states in various areas including supporting agencies to be assessed using the WHO Global Benchmarking Tool and to implement measures to address the gaps that have been identified.

### WHO Assessment of Regulatory Authorities

Various countries or jurisdictions have legislation mandating the regulation of medical products to ensure quality, safety, and efficacy [8]. The capacity to regulate medical products varies widely and traditionally, and countries that were members or observers of the International Council for Harmonisation of Technical Requirements for Pharmaceuticals for Human Use (ICH) were regarded as having stringent regulatory authorities (SRAs) [9]. However, the World Health Organization (WHO) has recently made a proposal to use the term WHO-listed authorities (maturity level 4) for authorities previously referred to as SRA and award-listed authority status to any additional authorities based on the Global Benchmarking Tool (GBTs) [9, 10]. This tool allows for the objective evaluation of national regulatory systems, as agreed by WHO Member states in the World Health Assembly Resolution 67.20 on Regulatory System Strengthening for medical products [11]. The GBT evaluates the overarching national regulatory system as well as the following functions that make up the regulatory system: registration and marketing authorization, market surveillance and control, regulatory inspection, vigilance, licensing establishments, clinical trial oversight, laboratory testing, and NRA lot release [10]. The WHO has begun the process of evaluating the regulatory systems of countries including low- and middle-income countries (LMICs). One of the outcomes of the assessments using the GBT is the development of an Institutional Development Plan, which identifies gaps as well as the activities and resources required to strengthen the regulatory system. As of May 2020, of the 55 countries in Africa, the WHO had benchmarked the national medicines regulatory agencies of 13 while 34 had conducted self-benchmarking, a pre-requisite for formal benchmarking by the WHO [12]. Tanzania and Ghana were benchmarked and attained maturity level 3 status which represents “a stable, well-functioning and integrated regulatory system” [9, 13, 14]. Regulatory reviews fall under the registration and marketing authorization function of the GBT.

Unlike high-income countries, there is limited information in the public domain on the regulatory review/assessment systems and performance of LMIC [15]. Evaluation of the regulatory review systems of a number of high-income and upper middle-income countries, for example, Saudi Arabia, Jordan, Turkey, and South Africa, are available in the literature [16–20]. However, it appears that there are few published assessments of the regulatory review systems in LMIC in Africa. The aim of this study was, therefore, to evaluate the current regulatory review process in Zimbabwe, identifying challenges and opportunities for growth and improvement.

## Study Objectives

The main objectives of this exploratory study were to:

1. Assess the current regulatory review process in Zimbabwe,
2. Identify the key milestones and target timelines in the review process,
3. Evaluate the overall performance for the review models and different product types approved in Zimbabwe during the period 2017 to 2019,
4. Evaluate how the quality of the process of decision making is built into the regulatory review process and registration of medicines, and
5. Identify the challenges and opportunities for an enhanced regulatory process in Zimbabwe, with a view to expediting patients’ access to life-saving medicines.

## Methods

### Ethical Approval

The authors’ institutions do not require ethics approval for the type of study reported here.

### Study Rationale

The study was planned as part of continuous improvement efforts of the agency as it was deemed important to identify the challenges and opportunities.

### Data Collection Process

A questionnaire technique [21] was used to identify the key milestones and activities associated with the review processes and practices within the MCAZ. The questionnaire was initially completed by a senior assessor, reviewed by the division’s management and verified by the Director General in 2019. To aid agencies who achieve the goals of regulatory efficiency, the Centre for Innovation in Regulatory Science (CIRS) developed a unique regulatory-strengthening tool entitled Optimising Efficiencies in Regulatory Agencies (OpERA). The OpERA project was initiated in 2013 based on requests from regulatory agencies, and the objectives of this program are to provide benchmarking data that can be used to define performance targets and focus ongoing performance improvement initiatives; accurately compare the processes used in the review of new medicines marketing authorizations; encourage the sharing of information on common practices in order to learn from others’ experiences; and encourage the systematic measuring of the processes that occur during the review of new medicines marketing authorization [22].

The questionnaire consists of 5 parts [21, 22].

*Part 1: Organization of the agency* documents the information on the structure, organization, and resources of the agency.

*Part 2: Types of review models* identify different types of review model(s) used for the scientific assessment of medicines in terms of the data assessed and level of detail by the agency, as well as how the agency might rely on the results of assessments and reviews carried out by a reference agency.

*Part 3: Key milestones in the review process* document information on the key milestone dates, using the online OpERA tool and map the process of assessment starting from receipt of the dossier, validation/screening, the number of cycles of scientific assessments including the questions to the sponsor/applicant and expert registration committee meetings to the final decision on approval or refusal of a product for registration. A standardized process map embedded in the questionnaire was based on the experience of studying established and emerging regulatory authorities. Data were collected for new chemical entities (NCEs), biologicals, and biosimilars, and generics registered by the Zimbabwean NRA during the period 2017–2019. These data were sourced directly from the division within the authority responsible for the regulatory review process.

*Part 4: Good review practices (GRevP)* evaluate how quality is built into the regulatory process by examining activities that have been adopted to improve consistency, transparency, timeliness, and competency in the review process.

*Part 5: Quality decision-making processes* explore the quality of agency decision-making practices and whether measures are in place to ensure that quality decisions are made around the data during the registration process.

### Models of Regulatory Review

There are three models for the scientific regulatory review of a product that can be used by regulatory authorities [21] and these are as follows:

(i) The verification review (type 1), which requires prior approval of a product by two or more reference or competent regulatory authorities allowing the agency relying on such assessments to employ a verification process to validate a product and ensure that it conforms to the previously authorized product specifications.
(ii) The abridged review (type 2), which involves an abridged evaluation of a medicine taking into consideration local factors and environment, with the pre-requisite of registration by at least one reference or competent regulatory authority.
(iii) The full review, type 3A, which involves the agency carrying out a full review of quality, safety, and efficacy, but requires that the product has previously been reviewed by an agency for which there is a CPP or type 3B which involves an independent assessment of a product’s quality, pre-clinical, as well as clinical safety and efficacy, but which has not been evaluated by any previous agency.

## Results

The results will be presented under five major headings: (Part I) organization of the agency (this section addresses objectives 1 and 5); (Part II) types of review models used in Zimbabwe (this section addresses objectives 1 and 5); (Part III) key milestones in the Zimbabwe Regulatory review process (this section addresses objectives 1, 2, 3, and 5; (Part IV) good review practices: building quality into the regulatory process (this section addresses objectives 1, 4, and 5); and (Part V) quality decision-making practices (this section of the results addresses objectives 1, 4, and 5).

### Part 1: Organization of the Agency

The MCAZ is an autonomous agency established in 1997 as a successor to the Drugs Control Council and the Zimbabwe Regional Quality Control Laboratory. The MCAZ regulates medicinal products for human and veterinary use as well as medical devices and diagnostics. The scope of control of medical devices is currently limited to gloves and condoms but will increase once the medical devices' regulations, which have been developed, are approved. The MCAZ scope of activities includes issuing of marketing authorizations/product licenses, post-marketing surveillance, laboratory analysis of samples, clinical trial authorization, regulation of advertising, site inspections/visits, import and export control, and licensing of premises and persons responsible for the manufacture, supply, distribution, storage, and sale of medicines.

The MCAZ currently has 143 full-time personnel including management, technical, and administrative staff. Eighteen full-time reviewers are dedicated to assess applications for marketing authorization/product licenses for synthetic and biological products, of whom 3 specialize in the review of biological products. As the MCAZ does not receive many applications for registration of biological products, the 3 reviewers also assess chemical/synthetic products (small molecules). The majority of the staff reviewing marketing authorization applications are pharmacists and some of them have post-graduate qualifications. However, no physicians are engaged in the regulatory review process for issuing marketing authorizations.

### Part 2: Types of Review Models Used in Zimbabwe

The MCAZ carries out all three types of established regulatory review [21], although there is some difference in the requirement of the number of approvals by a reference agency.

The verification (type 1) review is used only for WHO-prequalified (PQ) products through the WHO Collaborative Medicines Registration Procedure (CRP), typically foreign generic medicines [23]. This type of review is enabled because WHO shares unredacted assessment reports for PQ products with the manufacturer’s consent and WHO GMP inspection outcomes are also available. Reviews involve ensuring that the product approved by the WHO PQ is the same as that submitted to MCAZ and reviewing country-specific requirements such as labeling. Post-approval changes are communicated to the MCAZ by WHO PQ. The target timeline for this route is 90 calendar days (Table 1).

**Table 1.** Target Timelines for the MCAZ Review Process

Milestone/process	Target
Acknowledgement of receipt	30 calendar days
Screening/validation	60 calendar days
Acknowledgement/screening/validation	90 calendar days
Scientific assessment (per review cycle)	60 calendar days
Sponsor response time (per review cycle)	60 calendar days
Scientific assessment/sponsor response	120 calendar days
Expert Committee procedure	No target time
Authorization procedure	60 calendar days
Full review	480 calendar days
Abridged review	270 calendar days
Verification review (WHO CRP)	90 calendar days
Expedited review/fast track	180 calendar days
ZAZIBONA review	270 calendar days

The abridged (type 2) review is used for products approved by at least one reference authority; for example, the European Medicines Agency, Medicines and Healthcare Products Regulatory Authority, United States Food Drug Administration, Australian Therapeutic Goods Administration, Health Canada, Japanese Pharmaceuticals and Medical Devices Agency, and other mature agencies in Europe. This is the primary route for NCEs and biologicals. Generics and biosimilars approved by a reference agency will also go through the abridged route. However, the MCAZ does not have any formal agreements in place with any of these reference agencies to facilitate sharing of unredacted assessment reports; therefore, public assessment reports are used instead. The target timeline for this route is 270 calendar days (Table 1).

A full review (type 3A) of quality, safety, and efficacy is conducted for products not approved by any reference agency, and these products are usually generics and biosimilars. For generics, the chemistry, manufacturing and control (CMC), and bioequivalence are reviewed sequentially while for biosimilars the quality, non-clinical and clinical data are reviewed in parallel. The target timeline for this route is 480 calendar days (Table 1). ZaZiBoNa products undergo a full review; however, they are placed in their own queue with a target timeline of 270 days. A type 3B review which involves an independent assessment of pre-clinical (safety) and clinical (efficacy) data is not conducted.

An expedited/fast-track review is also conducted. Applications are placed at the front of the queue but can be assessed using any of the above types of review (1, 2, or 3) depending on the product. Applications from local manufacturing companies and products for unmet medical needs are also given a priority review. The target timeline for this route is 180 calendar days (Table 1).

#### Data Requirements and Assessment

At present, the Certificate of Pharmaceutical Product (CPP) is legally required for registration in Zimbabwe for all three review types, as this is used as evidence of registration in the country of origin and to confirm similarity of the product being submitted to Zimbabwe with the one that is approved in the country of origin. The requirement for the CPP may be waived at the time of submission of the application, but the CPP must be submitted prior to registration. Evidence of compliance with good manufacturing practices (GMP) for both the active pharmaceutical ingredient and finished pharmaceutical product manufacturers, product samples, copies of the labeling, and a full dossier (modules 1–5) is required for all review types. A detailed assessment of the data is carried out, and the relevant assessment reports are prepared. The MCAZ performs benefit-risk assessments during the abridged review of NCEs and biologicals, as well as during a full review of biosimilars taking into account differences in medical culture/practice, ethnic factors, national disease patterns, and unmet medical needs. As previously stated, the authority does not access internal assessment reports from other authorities except from the WHO through the collaborative registration procedure. However, publicly available reports such as European Public Assessment Reports and those from other reference/recognized agencies are used during the review process.

### Part 3: Key Milestones in the Zimbabwe Regulatory Review Process

**Figure 1.**
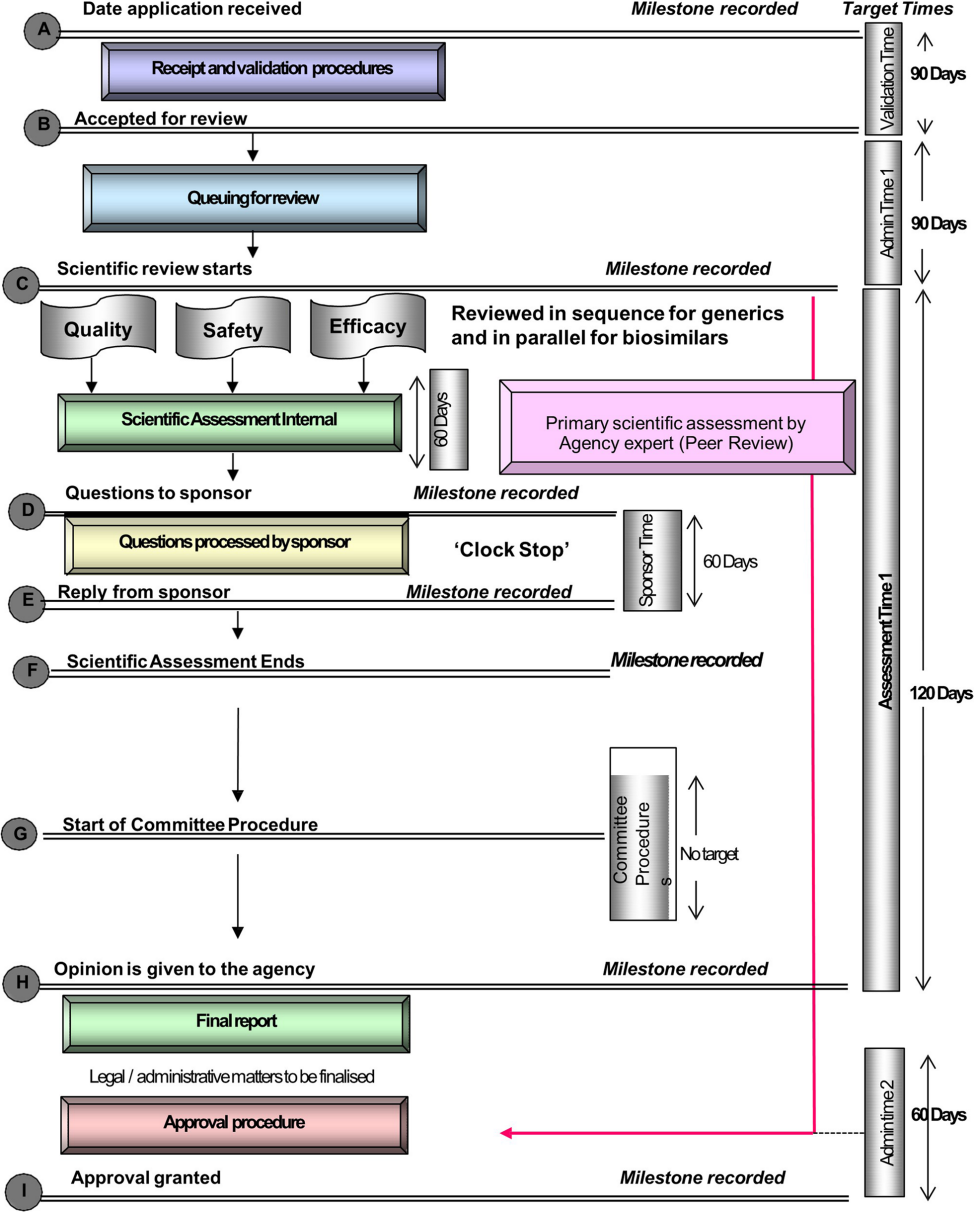
Regulatory Review Process Map for Zimbabwe Showing Target Times in Calendar Days. The Map Represents the Review and Authorization of a Product that Goes to Approval After One Review Cycle.

The regulatory review process and authorization of medicines are performed within the Evaluations and Registration division of the MCAZ, and this is depicted in Fig. 1 including milestones and timelines. This is a simplified representation of the main steps in the review of applications. The map represents the review and authorization of a product that goes to approval after one review cycle. In reality, it often takes a minimum of three review cycles before the review of a product is finalized. In addition, the map does not include steps such as the submission of representations to the administrative court within a specified period to appeal against the refusal of an application.

#### Receipt and Validation Procedures

All applications for registration are received by the Administrative Regulatory Officer and tabled before the Registration Committee. The target is to send an acknowledgement of receipt of the application by the committee within 30 calendar days from the date of receipt. Applications are then screened/validated and the target time for completion of this step is 90 calendar days from the date of receipt of the application. Products that fail screening are removed from the queue and applicant requested to provide the missing information. Products that pass screening are placed in a queue awaiting allocation to the next available assessor. The target time for start of the scientific assessment is 180 calendar days from the date of receipt of the application. All product types join the same queue and are assessed following the “first in first out” principle, regardless of the nature of the product or the review type, with the exception of expedited/fast-track review applications.

#### Scientific Assessment

The start of the scientific assessment is formally recorded. Scientific data are separated into quality, safety, and efficacy for review, and these are assessed sequentially by one assessor when it is a generic medicine. However, the sections may also be assessed in parallel by different assessors when it is a biosimiliar medicine. At present, the primary scientific assessment is carried out by the authority technical staff, although in the past, external assessors have been engaged under contractual agreement to work within deadlines set by the agency. Peer-reviewed assessment reports and recommendations are discussed by the external expert panel *Registration Committee,* which makes the final decision on registration or refusal of a product. The target timeline for each cycle of scientific assessment is 60 calendar days.

#### Questions to Applicant (Sponsor)

There is an opportunity for applicants to hold meetings with the agency staff to discuss questions and queries that arise during the assessment. A meeting record is generated during these meetings. Technical advisory meetings are also provided to local pharmaceutical manufacturers upon request; unlike other jurisdictions, no fee is charged for these meetings. Questions are collected into a single batch after each review cycle and only sent to the applicant after the Registration Committee has made its decision. The applicant is allowed 60 calendar days to respond after each review cycle; however, due to manual tracking and requests for extension to the deadline, company time can exceed this target time. The scientific review ceases while questions are being processed by the sponsor; that is, a *clock stop* is applied; however, this time is not excluded when median approval time is calculated in practice as well as in this study.

#### Expert Committees

The Registration Committee, which includes representatives from the disciplines of pharmacy, medicine, public health, toxicology, pharmaceutical science, biotechnology, and academia, meets once a month and makes decisions on registration or refusal of a product after the review of the scientific data by assessors. There is no target time limit for the Committee procedure. A letter communicating the Committee’s decision is prepared and questions communicated to the applicant/sponsor with a 60-day deadline. Responsibility for the decision lies with the Registration Committee, which uses a consensus process for decision making, and the MCAZ is mandated to follow its decisions. The criteria for granting or refusing a marketing authorization/registration relate only to the assessment of scientific data on quality, safety, and efficacy and is not dependent on a pricing agreement or on sample analysis. In some cases, sample analysis may be done in parallel with the scientific review, but for the majority of applications, the analysis is carried out post-registration. Information in the summary of product characteristics (SmPC) is reviewed, and for generics, this is expected to be similar to that of the reference/innovator SmPC. Compliance with local labeling requirements, e.g., pharmacological classification, is also a requirement for registration. Before a product is authorized, the manufacturing site must be deemed GMP compliant by the MCAZ inspectorate and this can be based on an onsite visit or a desk review where there is a GMP inspection by a recognized regulatory authority. The sponsor/applicant is informed of the authority’s intention to approve the registration as well as any conditions of approval before the authorization is issued. At that stage, the sponsor is given 30 calendar days to respond. It can take approximately 60 calendar days from receiving a positive scientific opinion and the intent to register decision to issuing an approval letter and certificate of registration (Table 1).

#### Approved Products and Review Times

##### Classification of Approved Products

**Figure 2.**
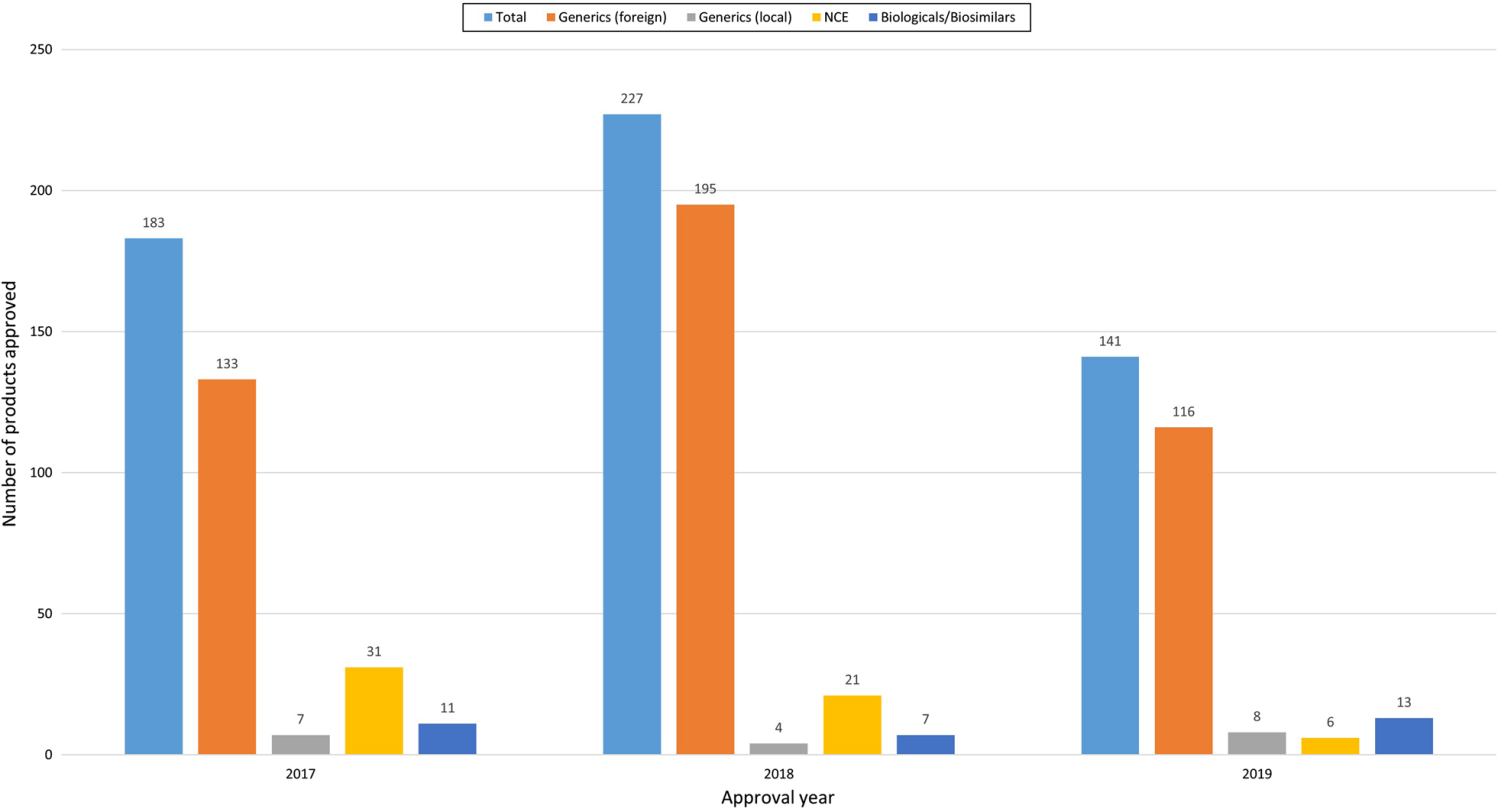
Number of Approved Products Classified into Total, Generics (Foreign), Generics (Local), New Chemical Entities, and Biologicals/Biosimilars.

From 2017–2019, 97% of approved products were submitted by foreign companies. The majority of applications approved during the study period were generics manufactured by foreign companies followed by NCEs, biologicals/biosimilars, and generics manufactured by local companies (Fig. 2). In 2017, 73% of the products approved were generics (foreign), 17% were NCEs, 6% were biologicals/biosimilars, and 4% were generics (local). In 2018, 86% of products registered were generics (foreign), 9% were NCEs, 3% were biologicals/biosimilars, and 2% were generics (local). In 2019, 82% of products registered were generics (foreign), 4% were NCE, 9% were biologicals/biosimilars, and 5% were generics (local). The highest number of products approved during the study period was 195 in 2018 for generics (foreign), 31 in 2017 for NCE, 13 in 2019 for biologicals/biosimilars, and 8 in 2019 for generics (local). There was a decreasing trend in the number of NCE approved over the study period. All approved NCEs were sponsored by foreign companies, there were no locally sponsored NCEs.

##### Review Times for Different Product Types

**Figure 3.**
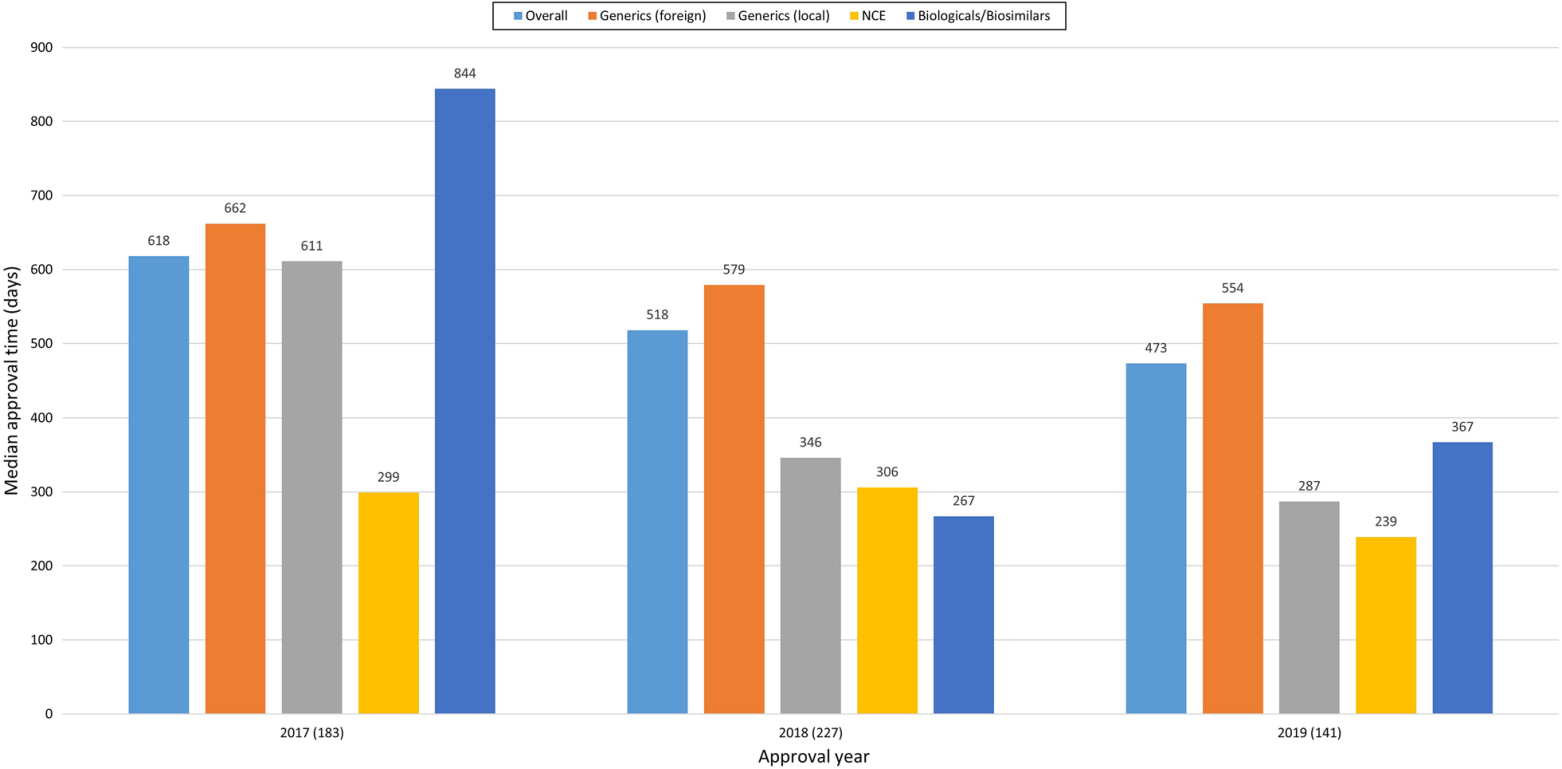
Median Approval Times (Inclusive of Applicants’ Time) for All Products (Overall), Generics (Foreign), Generics (Local), New Chemical Entities, and Biologicals/Biosimilars.

It is significant that there was an improvement in review times over the 3-year period for all categories of products. The median overall approval time for all products was reduced from 618 calendar days (*n* = 183) in 2017 to 518 days (*n* = 227) in 2018 and 473 days (*n* = 141) in 2019. The median approval time for generics (foreign) was reduced from 662 calendar days (*n* = 134) in 2017, to 579 days (*n* = 195) in 2018 and 554 days (*n* = 116) in 2019. The median approval time for local generics halved from 611 calendar days (*n* = 7) in 2017, to 346 days (*n* = 4) in 2018 and 287 days (*n* = 8) in 2019. The median approval time for NCEs has remained relatively constant at 299 calendar days (*n* = 31) in 2017, 306 days (*n* = 21) in 2018, and 239 days (*n* = 6) in 2019. The median approval time for biologicals/biosimilars was significantly reduced from 844 calendar days (*n* = 11) in 2017, to 267 days (*n* = 7) in 2018 and 367 days (*n* = 13) in 2019. The longest median approval time observed during the study period was (844 calendar days) for biologicals/biosimilars in 2017. The shortest median approval time observed was 239 calendar days for NCEs in 2019 (Fig. 3).

##### Comparison of Review Times for Different Models

**Figure 4.**
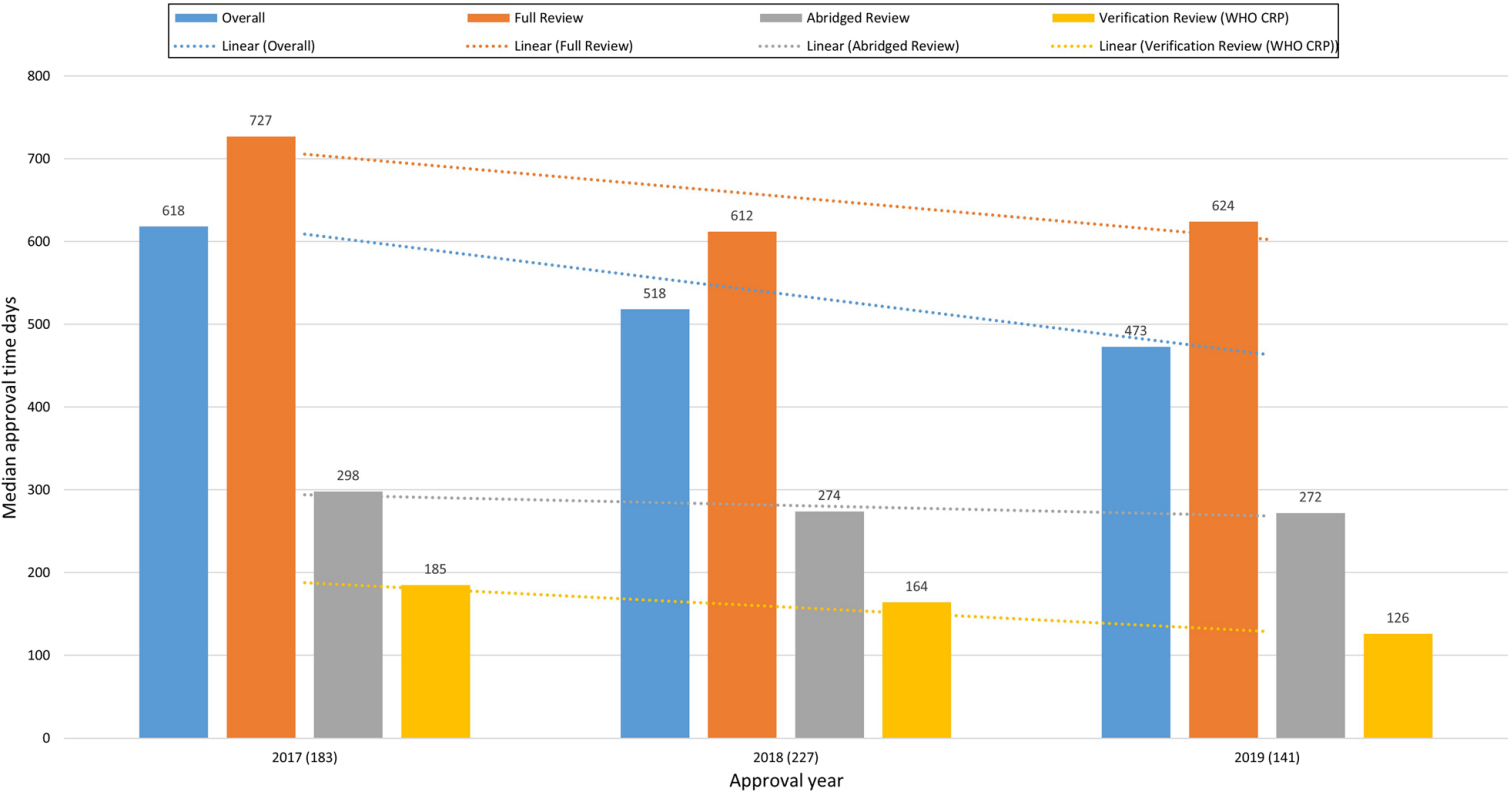
Median Approval Times (Inclusive of Applicants’ Time) for Different Review Models; i.e., Overall, Full Review, Abridged Review, and Verification Review (World Health Organization WHO Collaborative Medicines Registration Procedure).

An improvement in review times was observed across all review models over the three-year study period. The median approval time for full review (used for generics and biosimilars not approved by a reference authority) decreased from 727 days (*n* = 142) in 2017, to 612 days (*n* = 174) in 2018 and 624 days (*n* = 105) in 2019. The median approval time for abridged review (used for NCEs, biologicals, generics, and biosimilars approved by a reference authority) decreased from 298 days (*n* = 35) in 2017 to 274 days (*n* = 36) in 2018 and 272 days (*n* = 29) in 2019. The median approval time for verification review (used for WHO PQ products under the CRP) decreased from 185 days (*n* = 5) in 2017, to 164 days (*n* = 17) in 2018 and 126 days (*n* = 7) in 2019. The highest median approval time was 727 days (*n* = 142) in 2017 for products that had a full review, and the shortest was 126 days (*n* = 7) in 2019 for products that had a verification review (Fig. 4). In general, the median approval time for verification review was the shortest throughout the study period, followed by abridged review then full review. Products were approved in less than half the time taken for full review under abridged review and in approximately a quarter of the time under verification review for all three years.

### Part 4: Good Review Practices: Building Quality into the Regulatory Process

#### General Measures Used to Achieve Quality

GRevPs have been informally implemented by the agency, using WHO PQ as a standard, including the use of guidelines, standard operating procedures, assessment templates, and screening checklists (Table 2). These documents are not available to the public except the guidelines and the applicant’s screening checklist, which are available on the MCAZ website www.mcaz.co.zw. The MCAZ top management has endorsed and formally adopted an internal quality policy that gives direction related to the quality of the review process. The agency produces an assessment report in English, which undergoes a process of internal peer review before consideration by the Registration Committee. A Registration Committee preparatory meeting serves as a quality assurance check before reports are taken to the Committee. Applicants/sponsors do not get a full copy of the assessment report and a redacted assessment report is not published on the website. Other tools that build quality into the assessment process are: the availability of the following platforms for communicating with applicants/sponsors and obtaining their feedback; procedures for submitting complaints by applicants/sponsors; annual stakeholder meetings; individual client meetings; and liaison meetings with stakeholders such as association of pharmaceutical manufacturers, retail pharmacists, and pharmaceutical wholesalers.

**Table 2:**
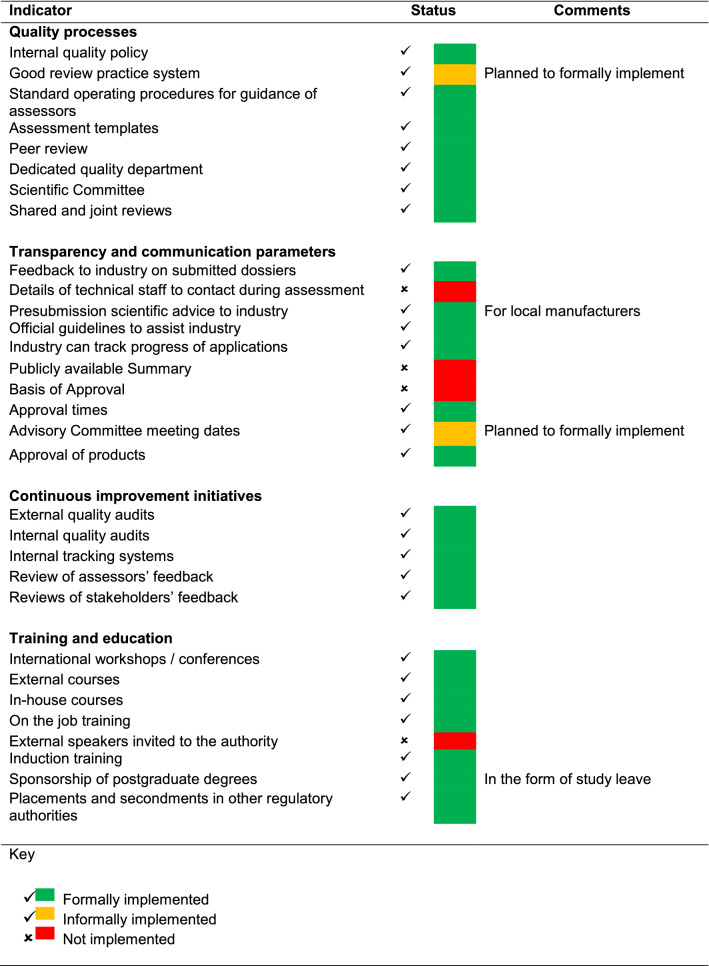
Status of implementation of good review practices by the MCAZ

#### Quality Management

The MCAZ has identified quality management to be critical in achieving its values which are customer focus, continuous improvement, integrity, and accountability. The authority strives to be more efficient, to ensure consistency, and to increase transparency. The following activities are undertaken to bring about continuous improvement in the assessment and authorization process: reviewing assessors’ feedback and taking necessary action; reviewing stakeholders’ feedback through, e.g., satisfaction surveys, complaints, meetings, or workshops and taking necessary action; using an internal tracking system to monitor quality parameters such as consistency, timeliness, efficiency, and accuracy; and carrying out internal quality audits such as self-assessments, as well as having external quality audits by accredited certification bodies and using the findings to improve the system. The authority has a dedicated Quality Unit for assessing and/or assuring quality in the assessment and registration process for medicines. Quality management review meetings are held quarterly to monitor implementation of quality standards across the organization.

#### Quality in the Review and Assessment Process

Some measures that have been implemented to help improve the quality of applications and the scientific review are publication of various guidelines to assist industry as well as regular feedback to applicants on common deficiencies observed in applications for registration. These are made available through the MCAZ website, industry associations, meetings with stakeholders, and upon request. In addition, pre-application scientific advice has been given mostly to local manufacturers/applicants. Quality is monitored through the minutes of such meetings. The applicant is not given the contact information of the assessor to discuss their application during the review. However, there is some formal contact to discuss the status of pending products. Meetings are held by appointment on specific days of the week; however, applicants can send emails at any time requesting status updates from the administrative regulatory officer. Phone calls are largely discouraged but may be taken on designated days.

#### Shared/Joint Reviews

The MCAZ is a founding member and active participant of the SADC collaborative medicines registration initiative ZaZiBoNA [3, 7]. The MCAZ acts as a rapporteur, performing the first review of a product application or as a co-rapporteur performing the peer review of an application for products assessed by the initiative for which marketing authorization in Zimbabwe is sought. The product application should have been submitted to a minimum of two countries to be eligible for review under ZaZiBoNa. The WHO carries out quality assurance for all reviews under the initiative. There are formal measures in place to ensure consistent quality during the review under the initiative through the use of guidance documents for assessors, use of common templates for assessment of generic medicines, and the availability of standard operating procedures. With the manufacturer’s consent, the agency shares the assessment report with other regulatory authorities for ZaZiBoNA products. The joint reviews have served as a platform for training, particularly assessment of the active pharmaceutical ingredient and biologicals/biosimilars as well as greater exposure to WHO standards of assessments. To date, ZaZiBoNa has contributed 11% of total registrations in Zimbabwe in 2017 and 2019, and 4% in 2018 [24].

#### Training and Continuing Education as an Element of Quality

A formal training strategy and program for assessors is in place which includes training at induction, on-the-job training, internal and external short courses, support for post-graduate degrees, placements/secondments to more established regulatory authorities such as WHO PQ and The Federal Institute for Drugs and Medical Devices (BfArM) in Germany, and mentoring of junior assessors by more experienced assessors including peer review. The MCAZ does not seek direct assistance of more experienced agencies for the development of SOPs and guidelines; however, guidelines published by more experienced agencies are referenced, adapted, or adopted during the development of country guidelines. The agency collaborates with other agencies in the training of assessors, e.g., during pre-assessment training sessions at ZaZiBoNA or as co-facilitators for courses offered under the MCAZ RCORE. The MCAZ participates in training offered by WHO and other agencies. Once completed, a system is in place to evaluate the impact of any given training on the individual and on the division. The MCAZ participated in the exercise to determine the level of competence of assessors using the WHO Global Competence Framework for Assessors together with other SADC countries.

#### Transparency of the Review Process

Being open and transparent in relationships with the public, professionals, and industry is in line with MCAZ organizational values and is of high priority**.** The MCAZ identified the following top three incentives for assigning resources to activities that enhance the openness of the regulatory system: political will, the need to increase confidence in the system, and the provision of assurance regarding safety measures. Measures to achieve transparency include the provision of details regarding the registration process on the MCAZ website including fees payable for the different pathways and regular stakeholder meetings to interact with applicants and discuss processes and timelines for approval. In addition, an online register of approved products is available on the website while approved, canceled, refused, and withdrawn products are periodically published in the Government gazette. Although the MCAZ does not share assessment reports with applicants, the listed deficiencies or questions raised during assessment are shared with the applicant, which they are given a period of 60 days to address. When a product is refused registration, the reasons for the refusal are shared with the applicant. Furthermore, detailed statistics are published in the annual reports which the Minister of Health and Child Care presents to the Parliament. Copies of the MCAZ Annual Reports from 2011–2018 are available on the MCAZ website. Customer satisfaction surveys and complaint forms, which are freely available on the website, are used to obtain feedback from applicants on the quality of the review process.

At present, it is not possible for companies themselves to track the progress of their applications; however, this is something that the authority plans to do in the future. However, companies can follow the progress of their applications through meetings, e-mail, and telephone contact. Currently, a database capable of archiving information on applications in a way that can be searched exists and an electronic tracking system has recently been implemented for internal use only.

### Part 5: Quality Decision-Making Practices

Although some good decision-making practices are implemented, the MCAZ does not have a validated documented framework in place that forms the basis of the decision to approve or reject an application. The current process in place is based on custom and practice. Assessors use a decision tree to assign relative importance, i.e., *critical* or *not critical* to findings, which ensures decisions/recommendations are made consistently regardless of the assessor.

One of the challenges identified is that the agency does not have measures in place to minimize the impact of subjective influences/biases on the agency’s decision making for the process to approve or reject an application. In addition, there is no training provided in the area of quality decision making in general and neither is there a formal assessment to periodically measure the quality of decision making within the agency for the process to approve or reject an application. There is, therefore, room for improvement of the authority’s decision-making process and the implementation of a framework.

## Discussion

The MCAZs vision is to be a leading and effective regulatory authority on the African continent. This is evidenced by its adoption of a robust quality management system and the implementation of good review practices in line with international best practice. Historically, the MCAZ has had the challenge of long registration times. Gwaza reported a range of 516 days to 1673 days median time to registration for the years 2003 to 2015 [15]. To address this challenge, the MCAZ invested in improving and re-engineering its processes using international standards as a benchmark. Management invested financially in the hiring of a dedicated administrative regulatory officer to perform validation of applications, thus, preventing incomplete applications from remaining in the pipeline. In addition, the hiring of dedicated dossier reviewers and the introduction of one-week off-site retreats allowed assessors to be dedicated to the review without any interruption. Management also invested in the development of an electronic tracking system, which triggered evaluation of the review process. This resulted in the setting of target times for all key milestones, adherence to target times, as well as stricter monitoring of deadlines given to applicants to respond to questions. The agency decided to limit the number of review cycles to three, which reduced the time spent with applicant addressing the same issues. Furthermore, the use of the abridged review model was extended to generics and biosimilars approved by recognized reference agencies, where previously it was only used for new chemical entities and biologicals. The results of this current evaluation show that the investment has been worthwhile as the regulatory review process now incorporates the milestones used by leading regulatory authorities globally, and therefore, this has led to a decrease in registration time. The improvement in the process has resulted in a decrease in the overall median approval time to 473 calendar days (15.8 months) in 2019, which is comparable to the review times of 10 to 16 months achieved for new active substances by mature and better resourced agencies [25]. The MCAZ has also shown initiative in using risk stratification approaches such as the abridged review pathway and participation in the WHO CRP. This has allowed the authority to focus its limited resources on the full review of applications for products that are not approved elsewhere.

### Performance Against Set Targets

The results of this study show that the authority is currently meeting the targets set for overall approval time (480 days) and abridged review (270 days). Although the time taken for approval using the verification review (WHO CRP) is above the target (90 days), it is still very short (125 days in 2019). The time taken for full review is much higher than the target of 480 days (624 days in 2019). Some of the reasons that contribute to a long approval time are a long queue time (the time a product spends in the queue from receipt to the start of the scientific assessment), an inadequate number of experienced reviewers, and numerous requests from applicants for deadline extensions to respond to reviewer questions. The queue time is indicative of the resources available to perform the work and a target of 180 days is too long, reflecting the need for an evaluation of the adequacy of human resources available to review products as well as the ability of the MCAZ to retain staff with key competencies and expertise. Gwaza reported that the authority had a relatively young workforce of assessors/reviewers, two of whom had doctorates at that time in 2014 [15]; however, when compared with results from the current study conducted five years later, the workforce is still relatively young and the two reviewers with doctorates are no longer a part of the team of reviewers. This points to a problem of high staff turnover and poor skills retention, which needs to be addressed if the queue time and overall timelines are to be improved.

### New Chemical Entities

While generics play an important and critical role in ensuring access to life-saving treatment in LMICs, the need for new and innovative medicines cannot be overlooked. Some patients have reported better outcomes with innovator brands compared with generic products [26], and NCEs should be approved and readily available on any market. This will reduce the cost of the medicine, unlike the situation in which the unregistered NCE is imported for the patient under section 75, a provision in the Medicines and Allied Substances Control Act, which waives the requirement for registration of unregistered medicines imported on a doctor’s prescription and named patient basis. NCEs or innovative products are normally only launched onto the African market after a number of years of approval and use in well-resourced markets [7] making them low-risk products with established efficacy and safety, which have undergone a rigorous review by a mature agency. The results of this study show that the MCAZ uses risk stratification for all NCEs by conducting an abridged review. This process has proven effective, as the median approval time for NCEs was the lowest of all the product types registered in Zimbabwe, ranging from 239 to 306 calendar days (8–10 months) over the study period, and this has not resulted in any increase in the incidence reports of post-marketing adverse events. The review times for NCEs are comparable to the time taken by mature agencies and much lower than the 3–6 years reported for review of new active substances in other countries in the region who conduct a full review [19, 20]. The results of this study show that all products are placed in the same queue for review regardless of the type of review to be conducted. This is different from some countries in the region where applications for NASs are placed in a different queue from applications for generic medicines [19]. There has been a decrease in the number of NCEs registered in Zimbabwe from 2017 to 2019 which could be due to various reasons, such as economic factors beyond the regulator’s control. However, the MCAZ can encourage submission or registration of NCEs by having a separate queue for these products since the numbers are very low compared with generics, and the type of review conducted is different. It is also likely that the NCEs will be addressing an unmet medical need. This will be a process improvement that will further reduce approval time and improve access to new and innovative life-saving medicines by patients in Zimbabwe.

### Biologicals and Biosimilars

The LMICs in the African region suffer the highest burden of infectious diseases such as HIV/AIDS and tuberculosis [27, 28], which has resulted in most of the countries developing policies to promote the prescription and use of generic medicines [29] to ensure access to treatment by as many patients as possible at affordable prices. In addition, in recent years, there has been a rise in the prevalence of non-communicable diseases such as cancer in LMICs [30, 31] and the cost of biologicals used for treatment of diseases such as cancer is prohibitive, leading to a rise in the use of biosimilar medicines. Review of applications for registration of biologicals and biosimilars requires different competencies to those required for small molecules. There is also a component of benefit-risk assessment to be considered for biosimilars that is not critical for small-molecule generic medicines.

From this study, we found that most biosimilars received in Zimbabwe require a full review as they are not approved by any of the reference authorities. The median approval time for biosimilars and biologicals of 844 calendar days (28 months) in 2017 was the highest for all product types during the study period. This was because in 2017, the agency had only just established a dedicated unit for biological products with three reviewers, and there was limited knowledge and experience to review these products. However, the greatest reduction in median approval time over the study period was observed for biologicals and biosimilars from 844 calendar days in 2017 to 267 days in 2018 owing to the reviewers gaining more knowledge and expertise in the area as well as the use of abridged review for biologicals and biosimilars approved by a recognized reference authority. A study should be conducted to determine why more manufacturers/applicants of biosimilars approved by reference authorities are not seeking market authorization for their products in the LMICs. Such products would drastically reduce the cost of treatment for patients who often have to pay out of pocket for treatment and therefore justifies a shorter registration times for such products.

### Local Products

Markets eroded by sub-standard and falsified medicines due to weak regulation, inadequate technology, outdated equipment and facilities, inadequate research and development, and lack of appropriately skilled personnel were cited as some of the challenges faced by the pharmaceutical manufacturers in Africa in the Pharmaceutical Manufacturing Plan for Africa (PMPA) business plan developed by a partnership of the African Union Commission (AUC) and the United Nations Industrial Development Organization [32]. The figures presented in this study on the number of generics registered from local and foreign companies, show that local manufacturers contributed 5%, 2%, and 6.5% respectively of the generic products registered from 2017–2019. Recognizing the role that local manufacturers can play in reducing the cost of medicines and contributing to public health, the MCAZ has recently adopted a policy to prioritize the review of locally manufactured medicines. This has resulted in a reduction in the median approval time (inclusive of the applicants’ time) of local generics from 611 calendar days (20 months) in 2017 to 346 days (11.5 months) in 2018, and 287 calendar days (9.5 months) in 2019. The MCAZ also plays a capacity-building role through the collaboration on the GMP roadmap for manufacturers and trainings offered to industry through its RCORE. It is envisaged that as the challenges identified in the PMPA business plan are addressed, the product portfolio of local manufacturers as well as their presence on the market will increase. The median approval time can be further reduced by limiting the number of review cycles and adhering to the deadlines for applicants to respond to questions (Figs. 3, 4).

### Electronic Tracking System

The MCAZ has recently implemented an electronic tracking system that should enable easier tracking and reporting of the *clock stop, clock start*. This will help both applicants/sponsors and the agency to see their contribution to the overall approval time. At present, the authority’s target timelines are set and measured inclusive of the applicant’s time. The shortcoming of this approach is that the authority includes company time when measuring its performance, yet this is not within its control. An element of good review practices yet to be implemented by the MCAZ is to enable applicants to track the progress of their applications. The authority should consider further improving the electronic tracking system to allow applicants to submit applications online and track their progress.

The MCAZ successfully implements the three types of review models in line with the international standards. The milestones in the review process are formally recorded, and targets have been set for each milestone. Performance against set targets is monitored. All except four indicators for good review practices are either formally or informally implemented. Although good decision-making practices are implemented, there is need to have a formal decision-making framework in place.

## Recommendations

The following opportunities for system/process improvement were identified from the study:

- The adequacy of human resources available to review products as well as the ability of the authority to retain staff with key competencies and expertise should be evaluated.
- The authority should consider mainly the agency time when setting target timelines and measuring performance and the timeframe for the applicant’s response should only be extended if there is a good rationale as this affects overall approval time.
- Applications should be placed in different queues according to review type, e.g., products requiring full review should have a separate queue from products eligible for abridged or verification review.
- The MCAZ should, where possible, pursue formal agreements with chosen reference agencies to facilitate the sharing of unredacted assessments reports or alternatively encourage manufacturers to use the recently published WHO collaborative procedure to facilitate the accelerated registration of products approved by mature regulatory agencies [33].
- The authority should consider improving the recently implemented electronic tracking system to allow applicants to track the progress of their applications in line with good review practices.
- Since there is no formal decision-making framework in place, the agency should implement a structured approach to decision making using a validated tool such as “Quality of Decision-Making Orientation Scheme (QDOS)” which identifies the 10 quality decision-making practices (QDMPs)
- The current templates and the benefit-risk framework used for abridged reviews should be evaluated and compared with those of comparable or reference agencies to determine if there is need for improvement.

## Conclusions

This study has evaluated the current MCAZ regulatory review process. Key milestones and timelines have been identified, and the measures used for GRevP have been considered. The MCAZ performs a full review assessment of applications for registration of generics and biosimilars not approved by a reference authority and uses reliance to conduct an abridged review for NCEs, biologicals, biosimilars, and generics approved by recognized reference regulatory authorities and verification review for WHO-prequalified products. A Quality Management System (ISO 9001) and quality policy are implemented. Overall, the results of this study demonstrated that the target timelines set and communicated by the authority to stakeholders are realistic and what is achievable with the current resources available. This transparency is commendable and enables applicants/sponsors to plan appropriately. The findings from this study present opportunities for an enhanced regulatory review and improvement of the current process. The study will enable the authority to easily identify the areas requiring additional resources and improvement. This study will also make it possible for comparison of Zimbabwe, a lower middle-income country, with similar countries in the SADC region, the African continent, and similar sized higher income countries beyond Africa with the goal to improve the regulatory review process in Zimbabwe and patients’ access to life-saving medicines. The approach taken here in this evaluation could also provide a model for other low- to middle-income countries in the African region.

## Abbreviations

AU: African Union
AUC: African Union Commission
AUDA NEPAD: African Union Development Agency New Partnership for Africa Development
AMRH: African Medicines Regulatory Harmonisation
CIRS: Centre for Innovation in Regulatory Science
CPP: Certificate of Pharmaceutical Product
CRP: Collaborative medicines registration procedure
GBT: Global Benchmarking Tool
GMP: Good manufacturing practice
GRevP: Good review practices
ICH: International Council for Harmonisation of Technical Requirements for Pharmaceuticals for Human Use
LMICs: Low- and middle-income countries
MASCA: Medicines and Allied Substances Control Act
MCAZ: Medicines Control Authority of Zimbabwe
MHRA: Medicines and Healthcare Products Regulatory Authority
MRH: Medicines registration harmonization
NRA: National regulatory agency
NCEs: New chemical entities
OpERA: Optimising Efficiencies in Regulatory Agencies
PMPA: Pharmaceutical Manufacturing Plan for Africa
QMS: Quality management systems
RCORE: Regional centre of regulatory excellence
SADC: Southern African Development Community
SOPs: Standard operating procedures
SRAs: Stringent regulatory authorities
SmPC: Summary of product characteristics
WHO: World Health Organization
